# Integrated personal health record (PHR) security: requirements and mechanisms

**DOI:** 10.1186/s12911-023-02225-0

**Published:** 2023-07-10

**Authors:** Azamossadat Hosseini, Hassan Emami, Yousef Sadat, Somayeh Paydar

**Affiliations:** 1 Health Information Management (HIM), Department of Health Information Technology and Management, School of Allied Medical Sciences, Shahid Beheshti University of Medical Sciences, Tehran, Iran; 2 Management of Technology, Department of Health Information Technology and Management, School of Allied Medical Sciences, Shahid Beheshti University of Medical Sciences, Tehran, Iran; 3 Health Information Management (HIM), Department of Health Information Technology, Faculty of Paramedicine, Hormozgan University of Medical Sciences, Bandar Abbas, Iran; 4 Health Information Management (HIM), Department of Health Information Technology, School of Paramedical Sciences, Kermanshah University of Medical Sciences, Kermanshah, Iran

**Keywords:** Security, Personal health record, Integrated PHR, personal health record security

## Abstract

**Background:**

Personal Health Records (PHRs) are designed to fulfill the goals of electronic health (eHealth) and empower the individual in the process of self-care. Integrated PHR can improve the quality of care, strengthen the patient-healthcare provider relationship, and reduce healthcare costs. Still, the process of PHR acceptance and use has been slow and mainly hindered by people’s concerns about the security of their personal health information. Thus, the present study aimed to identify the Integrated PHR security requirements and mechanisms.

**Methods:**

In this applied study, PHR security requirements were identified with a literature review of (library sources, research articles, scientific documents, and reliable websites). The identified requirements were classified, and a questionnaire was developed accordingly. Thirty experts completed the questionnaire in a two-round Delphi technique, and the data were analyzed by descriptive statistics.

**Results:**

The PHR security requirements were identified and classified into seven dimensions confidentiality, availability, integrity, authentication, authorization, non-repudiation, and right of access, each dimension having certain mechanisms. On average, the experts reached an agreement about the mechanisms of confidentiality (94.67%), availability (96.67%), integrity (93.33%), authentication (100%), authorization (97.78%), non-repudiation (100%), and right of access (90%).

**Conclusion:**

Integrated PHR security is a requirement for its acceptance and use. To design a useful and reliable integrated PHR, system designers, health policymakers, and healthcare organizations must identify and apply security requirements to guarantee the privacy and confidentiality of data.

**Supplementary Information:**

The online version contains supplementary material available at 10.1186/s12911-023-02225-0.

## Background

The Personal Health Record (PHR) is an electronic, lifelong resource of a person’s health information to make health decisions. Individuals own, organize and manage the information in the PHR, which adds by both the individual and their healthcare provider. This health information is shared in a private, secure, and confidential environment with the individual determining rights of access by the PHR owner [1, 2]. PHRs are robust health information technology tools that regard patients as active factors in the healthcare decision-making process [3, 4].

While there exists a variety of PHRs, there are three main categories of PHRs, which are as follows: (1) stand-alone or free-standing PHRs, which do not directly connect with any other systems and require manual data entry to populate and update the record. The most common types of these PHRs are either paper-based or personal computer-based; (2) tethered or institution-specific PHRs, which connect with the provider’s electronic medical record (EMR), with the web-based network and insurance company; (3) integrated or interconnected PHRs can be connected to multiple data networks and institutions [5–7]. The ideal type is an integrated PHR that can empower patients to manage their health care and facilitate continuous communication between patients and their health providers [8]. This type of PHRs can connect, exchange, and share information with a variety of information resources such as electronic health records (EHRs), insurance companies, pharmacies, and patients themselves [3, 9]. The PHR complements and is considered to be an element of the EHR and it is more comprehensive than the EHR as it includes information added by individuals such as diet and exercise routine [10]. The data in PHR is under the ownership and control of the patient, but in EHR it is under the ownership of healthcare providers [11]. By using safe and standard tools, patients and their families can integrate and manage healthcare information; as such, integrated PHRs are valuable assets for these groups [12]. Patients’ use of integrated PHR increases their awareness about healthcare, provides easier access to healthcare services, allows them to ask physicians questions, and helps them improve their health [13]. This electronic record empowers patients to self-manage their health, improves patient outcomes, decreases the cost of healthcare, enhances access to healthcare, especially in distance areas, and improves medication adherence [14]. Despite extensive efforts to increase patients’ access to their medical information through PHR in recent years, several legal, ethical, and technical challenges have seriously hindered PHR implementation [8, 15]. To promote PHR acceptance and ensure its successful implementation, it is thus essential to identify and elucidate factors that affect patients’ use of PHR [16]. Personal health information security is a major barrier to integrated PHR acceptance and usage [17–19]. The previous studies have discussed serious issues in implementing or using integrated PHR that is one of which was concern about the security of information [8, 20, 21].

Integrated PHR contains personal and health information that is sensitive information. Some people have concerns about storing and protecting this information online and consent to use them [8, 22]. Users’ trust in healthcare providers greatly depends on their awareness and perception of PHR privacy and security [23]. To ensure this trust, comprehensive security and privacy framework are needed to provide transparent regulations for access to, use, and disclosure of personal health information in PHR [24]. Perceived security and privacy have a positive influence on users’ attitudes and behavioral intentions in using integrated PHR to manage health information [25]. The establishment of private restrictions and security for information causes individuals to be able to control their personal information and guarantee its security and confidentiality [26].

According to the Markle Foundation’s Personal Health Technology Council, ensuring the security of information, respecting users’ privacy, and controlling their health records are essential to user acceptance of electronic information exchange and sharing integrated PHR [27]. Dimensions of data security and protection, including confidentiality, integrity, authentication, and availability should be included in PHR design and development for any activity that requires information storage and exchange [8, 26, 28]. As the breach of confidentiality and security of information poses an ethical barrier to the use of PHR, [28] the present study aimed to identify the requirements and mechanisms of integrated PHR security to guide PHR designers. In this article, we identified security requirements based on a literature review and categorized them into 7 dimensions that each dimension has different mechanisms. These requirements were confirmed by 30 experts in two rounds of the Delphi technique. For designing and implementing a reliable PHR, all identified security requirements and mechanisms should be considered.

## Methods

This applied study was developed via a two-stage process. In the first phase; to identify integrated PHR security requirements; a literature review was conducted by searching Web of Science, Scopus, PubMed, and Embase databases and websites of the American Health Information Management Association (AHIMA), the International Organization for Standardization (ISO), Health Level 7 (HL7). Then, security requirements were extracted from websites, online forms, and articles, and 7-dimensions of these requirements were selected based on the results of the literature review and research team view. A researcher-made questionnaire was designed based on the extracted requirements. The content validity of the questionnaire was confirmed by ten health information management and medical informatics experts.

**Fig. 1:**
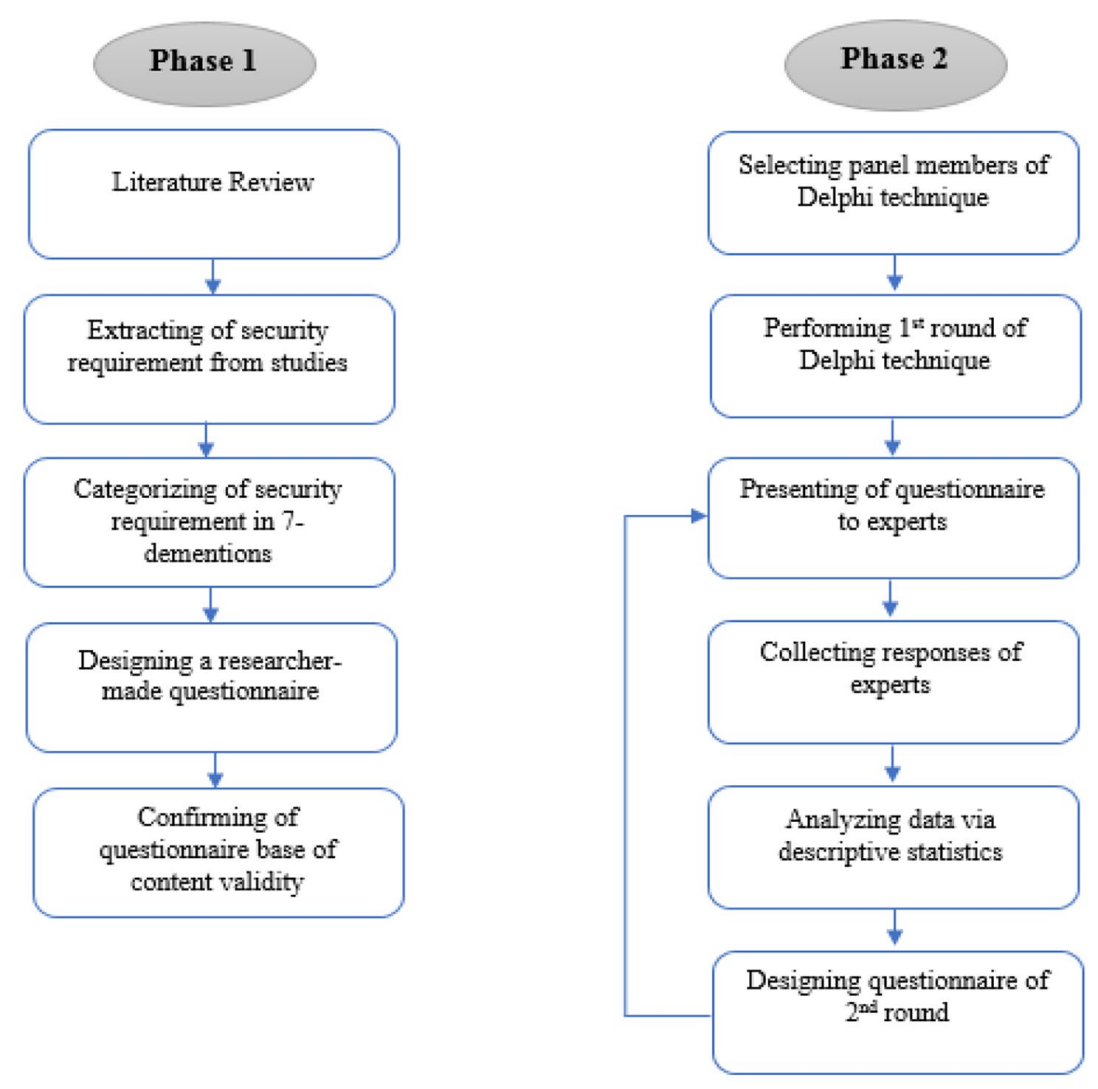
Diagram of the research method phases

In the second phase, the research team selects a group of experts based on the study topic as panel members of the Delphi technique. The selection of these experts was done by purposive random sampling method. Experts were Faculty members of health information management (10 experts) and medical informatics (10 experts) of Medical Sciences Universities with at least 5 years of experience as academic staff and experts or officials of the Statistics and Technology Information Management Center (10 experts) of the Ministry of Health and Medical Education with 5 years of experience in the field of electronic health records and information technology projects. Then, two rounds of questionnaires are presented to experts, and responses are aggregated after each round. Collected responses after each round were analyzed via descriptive statistics (number and frequency percentage) in Microsoft Excel 2019. All the questions with a score of > 75% achieved expert consensus, all the questions with a score of 50–75% entered the second Delphi round, and the questions with a score of < 50% were eliminated in the first Delphi round. As such, another questionnaire was designed for the second Delphi round to apply the comments and modifications of the first round (Fig. 1).

## Results

Seven dimensions were determined based on the first phase finding of the study that were obtained through the literature review. These seven dimensions were classified into confidentiality, availability, integrity, authentication, authorization, non-repudiation, and access rights. Each dimension comprised certain mechanisms as shown in Table 1.

**Table 1:** Security Requirements and Mechanisms of integrated PHR

Requirements	Mechanisms
**Confidentiality**	• Registering authorized PHR users • Determining the information sensitivity level in PHR • Encrypting data or key fields in PHR databases • Hiding information from unauthorized users • Restrictions of information updating by unauthorized users
**Availability**	• Creating an information backup • Specifying data access control list
**Integrity**	• Using a digital signature • Determining the standard terminology
**Authentication**	• Assigning user ID to all users • Determining password mechanisms • Using biometric scans (fingerprints, face, hands, retina)
**Authorization**	• Defining the roles (patient, provider, system manager, etc.) • Defining users’ access level to information • Compiling user’s list to access information in emergencies
**Non-repudiation**	• Creating an audit log (information audit) • Creating users’ accountability for any changes and manipulations
**Access right**	• Determining the time and individual (authorized users) to access personal health data by PHR owner • Authorizing another user to access & control the information for sharing by PHR owner • Reviewing entities’ access to personal health data by PHR owner • Revocation of entities’ access by PHR owner at any time • Restricting the previous physician’s access right to PHR

The findings of the first Delphi round indicate that the suggestions about PHR security requirements achieved an expert agreement of > 76% (Table 2).

**Table 2:** 1st Round of Security Requirements and Mechanisms of Integrated PHR

Security requirements & mechanisms		Experts Opinions			
		**Agree**		**Disagree**	
		**Number**	**Percent**	**Number**	**Percent**
Confidentiality	Registering authorized PHR users	28	93.33	2	6.67
	Determining the information sensitivity level in PHR	30	100	0	0
	Encrypting data or key fields in PHR databases	30	100	0	0
	Hiding Information from unauthorized users	30	100	0	0
	Restrictions of information updating by unauthorized users	24	80	6	20
Availability	Creating an information backup	28	93.33	2	6.67
	Specifying data access control list	30	100	0	0
Integrity	Using digital signature	26	86.67	4	13.33
	Determining the Standard terminology	30	100	0	0
Authentication	Assigning username to all user	30	100	0	0
	Determining Password mechanisms	30	100	0	0
	Using biometric scans (fingerprints, face, hands, retina)	30	100	0	0
Authorization	Defining the roles (patient, provider, system manager, etc.)	30	100	0	0
	Defining users’ access level to information	28	93.33	2	6.67
	Compiling user’s list to access information in emergencies	30	100	0	0
Non-Repudiation	Creating an audit log (information audit)	30	100	0	0
	Creating accountability of users for any changes and manipulations	30	100	0	0
Access Right	Determining the time and individual (authorized users) to access personal health data by PHR owner	28	93.33	2	6.67
	Authorizing another user to access & control the information for sharing by the PHR owner	28	93.33	2	6.67
	Reviewing entities’ access to personal health data by PHR owner	30	100	0	0
	Revocation of entities’ access by PHR owner at any time	23	76.67	7	23.33
	Restricting the previous physician’s access right to PHR	26	86.67	4	13.33

Among confidentiality requirements, “Restrictions of information updating by users unauthorized” received the lowest score with 6 “disagree”. Availability and authorization requirements (> 93.33%) and integrity requirements (> 86.67%) also achieved expert agreement. The experts also confirmed all the authentication and non-repudiation requirements (100%). Among the right of access requirements, “Revocation of entities’ access by PHR owner at any time” had the lowest score with 7 “disagree”, while the rest of the items attained > 86% agreement.

The experts suggested “using reCAPTCHA to prevent bots from logging into the system” (authentication dimension) in the first Delphi round, and this item attained 100% agreement in the second Delphi round.

## Discussion

This study discusses the security requirements of integrated PHR. Nowadays, a bidirectional flow between PHR and EHR systems is increasing for health care information exchange. Integrated PHRs combine EHR information from institutional medical records with patient self-reported data [29, 30]. A common concern for Designing of integrated PHR is information security and patient privacy [31, 32]. According to Harahap et al. study, the information security of integrated PHRs is ensured by mechanisms such as a single sign-on mechanism, authorization, user authentication, encryption or pseudonymization, backup mechanism, identity verification, and firewalls [8]. in our study. the dimensions of confidentiality, integrity, availability, authentication, authorization, non-repudiation, and right of access were identified as PHR security requirements. Mathuria et al. [33] and Israelson et al. [34] reported the security and right of access requirements to include patient information confidentiality, data integrity, authentication, authorization, non-repudiation, right of secure access to information, and access to information in emergencies. These findings are consistent with the current study.

Confidentiality as one of the security requirements assures that only authorized users can access PHR information and, as such, is a fundamental security requirement for sensitive PHR data. No unauthorized user should access PHR information unless authorized by the PHR owner [35]. The study results of Padol et al. indicated that patients are worried about unauthorized access, hacking, and lack of trust regarding their personal health information. They proposed solutions including HIPAA regulation, encryption and decryption, time stamp and control access [36]. The HIPAA regulations mandate that patients should have the right to access and receive a copy of their PHR. In addition, all healthcare systems (EHR, PHR, etc.) must adhere to HIPAA regulations, including the security, privacy, transfer, and release of patients’ medical information; and patients should be able to consent to and authorize the sharing of their PHR data with EHR systems [31]. Also, other studies provide results in line with this study that confidentiality requirements and data integrity are earned through data encryption, hiding and anonymization [34, 37–40], registering authorized users (healthcare providers or other patient-designated users) [41], determining confidential information and the information sensitivity level in the PHR [42].

An essential feature of integrated PHR is adding the ability to import and export full or partial backups [43]. According to Harahap et al. [8], Coatrieux et al. [44], and Zhou et al. [45], a backup option as an availability mechanism can avoid data loss and provide audit logs to review what data have been accessed and who accessed them.

Integrity ensures that unauthorized users cannot manipulate PHR data [35]. According to the HIPAA security regulations about integrity, covered entities must formulate policies and take measures to protect personal health data against inappropriate manipulation or destruction [46]. Digital signing is a very useful tool for ensuring the accuracy and integrity of data [47, 48] The use of terminology systems when exchanging data also ensures integrity [27, 49]. Both these mechanisms achieved expert agreement as integrity requirements in the present study.

To improve security and privacy, PHRs should implement access control, which includes authentication and authorization [8]. Authentication and authorization are other methods to assures the security of information systems. Authentication ensures that no unauthorized user can log into the system, and authorization guarantees that no user can access unauthorized resources by mistake [50, 51]. The most common authentication mechanisms in health records include the use of usernames and passwords [52] that achieved agreement in the present study, along with biometric scans (fingerprints, face, hands, retina).

Dimitropoulos [53] and HIPAA [54] mention “defining access levels and the role of authorized users to access information” as an authorization mechanism. Herein, this mechanism and “compiling a list of users who can access information in emergencies” were confirmed by experts. Chaudhary et al. noted that non-repudiation prevents users from denying that they have accessed or manipulated documents [35]. The National Committee on Vital and Health Statistics (NCVHS) hails audit log creation as a principle of non-repudiation [34]. According to Dalglish and Archer, the audit function is essential to compile a list of users who have accessed PHR data, so that unauthorized defects in information can be detected [23]. Similarly, in the current study, “creating an audit log” and “users’ responsibility for any modification or manipulation” achieved expert agreement.

In PHRs, patients have the right to control their data and authorize access to/addition of information. The owner of data can authorize or reject access to all/part of the data to all/some users [31]. In line with this study, the Markle Foundation’s Personal Health Technology Council declares that owners should have the right to assign the users who can access their PHRs, set the time of access, authorize other individuals to control this access and sharing of information, and view different entities’ access to their information [27]. Also, Park et al. stated providers can obtain information from PHRs only when authorized through access controls set by the PHR owner [55].

## Conclusion

In integrated PHRs, people can access and control their health information at any time, from any place, and on any computer to participate in their healthcare. The wide adaption and implementation of PHR can confer advantages, e.g., reducing healthcare costs, improving the quality of care, and achieving better health outcomes. The two distinct groups who have the greatest interest in creating and maintaining Personal Health Records (PHRs) are consumers (patients and their caregivers or healthy individuals) and healthcare providers (physicians or hospitals). Other stakeholders who have a stake in PHRs may include payers, employers, organizations, government, and health insurance companies. But security and privacy concerns have a seriously negative effect on the use intention of PHR by these stakeholders. The security requirements and mechanisms identified in this study can be used by system designers, health policymakers, and healthcare organizations to design a reliable PHR. These requirements can be used in future studies to develop, implement and evaluate health records and information systems. Also, modern technologies are used to achieve these requirements which can be the subject of future studies.

## Electronic supplementary material

Below is the link to the electronic supplementary material.

Supplementary Material 1

## Data Availability

The data used and/or analyzed during the current study are available from the corresponding author upon reasonable request.

## Abbreviations

PHRs: Personal Health Records
EHR: Electronic Health Record
HIPAA: Health Insurance Portability and Accountability Act
AHIMA: American Health Information Management Association
ISO: International Organization for Standardization
HL7: Health Level 7
NCVHS: National Committee on Vital and Health Statistics
