# Supplementary material for: Integrated personal health record (PHR) security: requirements and mechanisms
Source: BMC Med Inform Decis Mak. 2023 Jul 10;23:116. doi: 10.1186/s12911-023-02225-0 (PMC10334660; doi:10.1186/s12911-023-02225-0)
Supplement: Supplementary file 1 — Supplementary Material 1 [file 12911_2023_2225_MOESM1_ESM.docx]

**Appendix A: Questionnaire of Integrated Personal Health Record (PHR): Requirements and Mechanisms**

| **Confidentiality Requirements** | **Agree** | **Disagree** |
| --- | --- | --- |
| Registering authorized PHR users is one of the mechanisms to maintain confidentiality. | 🞎 | 🞎 |
| Determining the information sensitivity level in PHR is one of the mechanisms to maintain confidentiality. | 🞎 | 🞎 |
| In order to guarantee confidentiality, data or key fields in PHR databases must be encrypted. | 🞎 | 🞎 |
| Hiding information from unauthorized users is one of the mechanisms to maintain confidentiality. | 🞎 | 🞎 |
| In order to ensure confidentiality, information updating by unauthorized users should be restricted. | 🞎 | 🞎 |
| Please state any suggestions for confidentiality requirements for integrated PHR.  …………………………………………………………………………………………………………….…………………………………………………………………………………………………… | | |

| **Availability Requirements** | **Agree** | **Disagree** |
| --- | --- | --- |
| Creating an information backup leads to the availability PHR. | 🞎 | 🞎 |
| Specifying data access control list is one of the mechanisms of information availability. | 🞎 | 🞎 |
| Please state any suggestions for availability requirements for integrated PHR.  …………………………………………………………………………………………………….…  …….………………………………………………………………………………………………… | | |

| **Integrity Requirements** | **Agree** | **Disagree** |
| --- | --- | --- |
| Using a digital signature is one of the data integrity mechanisms. | 🞎 | 🞎 |
| Determining the standard terminology is one of the data integrity mechanisms. | 🞎 | 🞎 |
| Please state any suggestions for integrity requirements for integrated PHR.  ……………………………………………………………………………………………….………  …….………………………………………………………………………………………………… | | |

| **Authentication Requirements** | **Agree** | **Disagree** |
| --- | --- | --- |
| In order to ensure authentication, it is necessary to assign user ID for all users. | 🞎 | 🞎 |
| Determining password mechanisms is one of the authentication mechanisms. | 🞎 | 🞎 |
| The use of biometric scans (fingerprints, face, hands, retina) is one of the authentication mechanisms. | 🞎 | 🞎 |
| Please state any suggestions for authentication requirements for integrated PHR.  …………………………………………………………………………………………………….…  …….………………………………………………………………………………………………… | | |

| **Non-repudiation Requirements** | **Agree** | **Disagree** |
| --- | --- | --- |
| Creating an audit log (information audit) is one of the non-repudiation mechanisms. | 🞎 | 🞎 |
| Accountability of users for any changes and manipulations is one of the non-repudiation mechanisms. | 🞎 | 🞎 |
| Please state any suggestions for non-repudiation requirements for integrated PHR.  …………………………………………………………………………………………………….…  …….………………………………………………………………………………………………… | | |

| **Authorization Requirements** | **Agree** | **Disagree** |
| --- | --- | --- |
| Defining the roles (patient, provider, system manager, etc.) is one of the authorization mechanisms of PHR. | 🞎 | 🞎 |
| Defining users’ access level to information is one of the authorization mechanisms of PHR | 🞎 | 🞎 |
| Compiling user’s list to access information in emergencies is one of the authorization mechanisms of PHR | 🞎 | 🞎 |
| Please state any suggestions for authorization requirements for integrated PHR.  …………………………………………………………………………………………………….…  …….………………………………………………………………………………………………… | | |

| **Access right Requirements** | **Agree** | **Disagree** |
| --- | --- | --- |
| PHR owner can determine when and who (authorized users) can access his/her personal health data. | 🞎 | 🞎 |
| PHR owner can authorize another user to access & control the information for sharing them. | 🞎 | 🞎 |
| PHR owner can review entities’ access to personal health data. | 🞎 | 🞎 |
| PHR owner can evocate of entities’ access right to their PHR at any time. | 🞎 | 🞎 |
| PHR owner can restrict the previous physician's access right to PHR. | 🞎 | 🞎 |
| Please state any suggestions for access right requirements for integrated PHR.  …………………………………………………………………………………………………….…  …….………………………………………………………………………………………………… | | |
